# GCOD - GeneChip Oncology Database

**DOI:** 10.1186/1471-2105-12-46

**Published:** 2011-02-03

**Authors:** Fenglong Liu, Joseph A White, Corina Antonescu, Daniel Gusenleitner, John Quackenbush

**Affiliations:** 1 Monsanto Company, 8520 University Green, Middleton, WI 53562, USA; 2 Department of Biostatistics and Computational Biology, Dana-Farber Cancer Institute, 450 Brookline Ave., Boston, MA 02215, USA

## Abstract

**Background:**

DNA microarrays have become a nearly ubiquitous tool for the study of human disease, and nowhere is this more true than in cancer. With hundreds of studies and thousands of expression profiles representing the majority of human cancers completed and in public databases, the challenge has been effectively accessing and using this wealth of data.

**Description:**

To address this issue we have collected published human cancer gene expression datasets generated on the Affymetrix GeneChip platform, and carefully annotated those studies with a focus on providing accurate sample annotation. To facilitate comparison between datasets, we implemented a consistent data normalization and transformation protocol and then applied stringent quality control procedures to flag low-quality assays.

**Conclusion:**

The resulting resource, the GeneChip Oncology Database, is available through a publicly accessible website that provides several query options and analytical tools through an intuitive interface.

## Background

Although gene expression microarrays have been widely used to study human disease, by far the most extensive application has been to the analysis of human cancers. Despite the large number of array experiments deposited in public databases such as GEO [1] and ArrayExpress [2], our ability to perform meta-analyses of these data to discover cross-cutting patterns has been hampered by both the heterogeneous nature of the data and the lack of consistent annotation of the experimental samples. Although there have been some attempts to organize these data in resources such as Oncomine [3] and Genevestigator [4], both focus on analyses of subsets of the data and neither fully addresses the problem of integration across studies.

To overcome these limitations, we developed GCOD, the GeneChip Oncology Database, a freely-available web-accessible resource focused on gene expression profiles in cancer collected on the Affymetrix GeneChip platform. Relative to other resources, GCOD has three distinguishing features that we believe greatly enhance its overall utility. First, since GCOD focuses on expression data derived from a single platform and on studies where raw data are available, all datasets in GCOD are uniformly processed and properly scaled such that levels of gene expression in multiple samples across studies are comparable. Second, quality control protocols have been implemented in GCOD so that samples from hybridizations of questionable quality are identified and removed, improving the reliability of any subsequent data analysis. Third, and most importantly, sample annotations are manually curated based on descriptions in the paper and provided in a tabular format that is compatible with most microarray data analysis packages.

GCOD has a number if advantages over other databases. First, the data have been reprocessed to provide normalized and scaled values that can be compared across studies. This is not possible at GEO as it is simply an access portal that has no online analysis tools. Although ArrayExpress has several basic analysis tools, the data are not consistently normalized, making global analyses and their interpretation difficult. Oncomine provides access to a variety of data types, but access is limited for non-paying users so that certain data are not available. Genevestigator is freely accessible to academic users, but places access limitations on result sets and analysis tool access for those who have not paid. Genevestigator only includes data from GEO and not those in ArrayExpress. In contrast, GCOD contains a more comprehensive collection of cancer data, available without restriction, and includes a set of basic analysis tools.

## Construction and Content

### Data processing

Raw data (CEL) files from experiments run on the Affymetrix GeneChip platform are identified based on the keyword "cancer" and downloaded from public databases. These were first processed using the MAS5.0 algorithm (mas5) implemented in the Bioconductor package 'affy' to get detection calls and the 3' to 5' signal ratios for the GAPDH and β-ACTIN probesets. All the CEL files were then normalized using RMA [5,6] (rma in the affy package) for each experimental group (study). After RMA normalization, expression values are scaled such that the mean of each experiment is set equal to a common value.

For each GeneChip platform, probset definitions and other annotation are obtained from CDF (chip description files) files, supplied by Affymetrix,

Sample information accompanying source data files are parsed and manually curated using information in the accompanying publication to classify samples based on experimental factors including primary tissue source, cancer status (cancer or normal), primary tumor or metastasis, and treatment. Processed data along with detailed sample information were loaded into our GCOD relational database. A summary of all datasets available is listed in Table 1 (cancer types and number of hybridizations), and details about each study are listed in Additional File 1 (the entire list of studies; GEO or AE accession; PubMed references; number of samples); this is also available from the GCOD website (http://compbio.dfci.harvard.edu/gcod).

**Table 1:** Number of Arrays in GCOD Grouped by Cancer Type

Cancer Type	Number of Arrays
adrenal cancer	50
bladder cancer	234
brain cancer	818
breast cancer	2789
cancer cell lines	950
cervical cancer	33
colon cancer	73
endometrium	18
esophagus cancer	24
germ cell cancer	214
head and neck cancer	197
kidney cancer	40
leukemia	1760
lung cancer	560
lymphoma	358
multicancer	511
multiple myeloma	192
nci60	478
normal tissue	120
ovarian cancer	277
pancreas cancer	78
prostate cancer	952
renal cancer	34
sarcoma	60
thyroid cancer	96
Unassigned	2681

Total	13597

Counts of the numbers of arrays stored in the GCOD as a function of assigned cancer type.

### Database implementation

**Figure 1:**
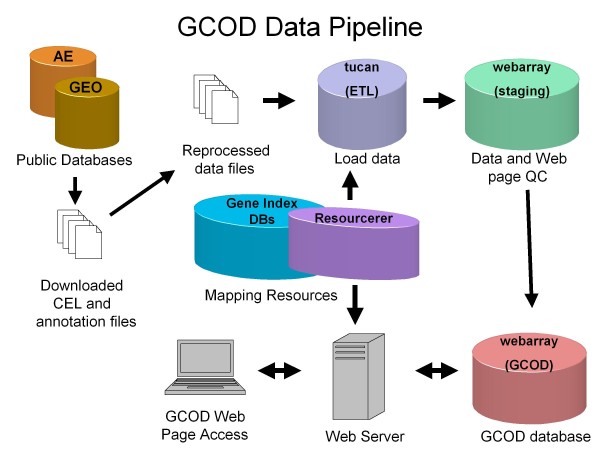
**Schematic representation of the GCOD databases**. Publicly available gene expression data are downloaded from ArrayExpress or GEO. These CEL and sample annotation files are reprocessed and saved as flat files; the MAS5 normalized, RMA normalized, scaled-RMA expression data, and curated sample annotation data are loaded into an ETL database having a schema in 3^rd^ normal form. There the data are further curated, and then transferred to a QA/QC database having a warehouse schema. In the QA/QC database the data are viewed on our internal web site to assess completeness. The data are then transferred to our GCOD database schema, which is accessed by the GCOD web application. Translation of GenBank and probeset identifiers is done by querying the TGI Resourcerer databases.

The GCOD database is implemented in an Oracle database system that consists of 3 separate database servers and an Apache web server that hosts the GCOD web site (Figure 1).

#### Database Implementation

After curation and normalization, data are loaded into an ETL (extract, transform and load) database via a series of Perl scripts designed specifically for the formats produced during curation. The ETL database is used for loading and cleaning the data prior to transfer to a QA/QC database; the schema for the ETL database is normalized to 3rd normal form. Oracle sqlldr is used to bulk-load gene expression data from the flat-files written by the Perl scripts into target database tables. These data are marked with a 'data set' identifier (dba_id), so that the results of an analysis can be rapidly accessed. Once the data for a study are completely loaded and checked, they are transferred from the ETL database to our QA/QC database by SQL insert statements issued on an Oracle database link. The QA/QC database is the data source for our internal web site, which we use to evaluate the presentation and completeness of the data in the web pages generated by our Apache server. After inspection, the data are transferred to the production GCOD database by SQL insert statements issued on an Oracle database link.

#### Data Model

**Figure 2:**
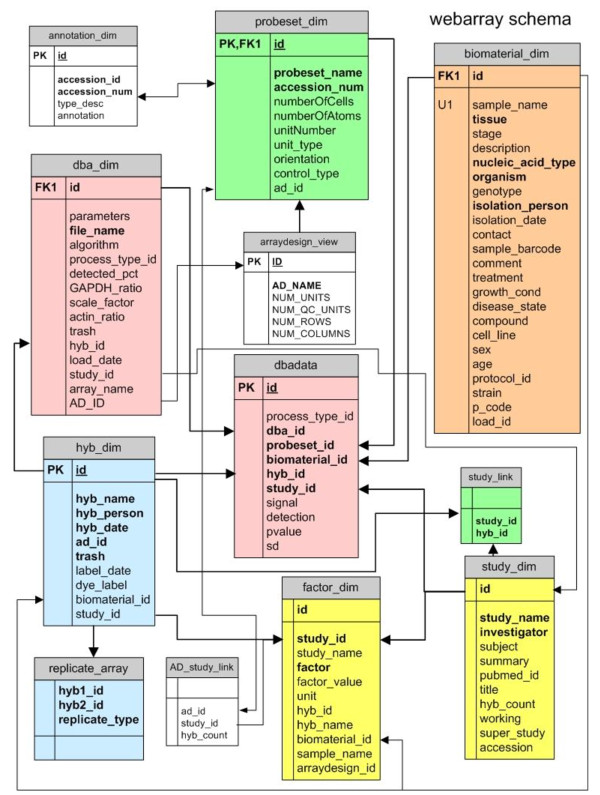
**GCOD Database Schema**. The schema for the GCOD database consists of 12 tables in a "star" layout. The main 'fact' table, dbadata, is split into 3 tablespaces that are distinguished by the algorithm used to generate the data, either MAS5, RMA, or scaled RMA, resp. The remaining tables are 'dimension' tables that contain characteristic and attribute information about the objects in the database. Lines and arrows indicate the relationships between tables and the key field linking the tables together.

The production GCOD database is maintained in an Oracle 11 g database server, which includes multiple schemas, in addition to the GCOD schema (Figure 2). The schema for this database follows a "star" schema, dimensional database model, in which measurement data (such as expression data values) are stored in a "fact" table, and categorical data are stored in "dimension" tables. The dimensions represent items that include studies, experimental factors, probesets, sample materials, array designs, hybridizations, and data sets.

The main fact table, dbadata, is partitioned into 3 separate tablespaces based on the analysis algorithm used to generate the data (either MAS5, RMA or RMA + scaling) and indexed on those foreign keys most commonly used in our queries. This produces exceptionally fast retrieval times. The dimension tables are not partitioned. The GCOD web site relies on information provided by the Gene Index (TGI) Resourcerer database which also resides in the production database server. The TGI database supplies GenBank accessions, PubMed identifiers and pre-computed mappings between various Affymetrix chips.

#### Web site implementation

The GCOD website is implemented on an Apache web server through a series of Perl/CGI/DBI scripts. These scripts use the CGI interface to present web pages, and the DBI interface to access and query the production GCOD database. The Perl/CGI/DBI scripts also access Resourcerer to obtain pre-computed mappings between Genbank, Ref_seq, probeset and array-type identifiers. This allows us to map probesets on one Affymetrix chip to probesets on another Affymetrix chip (whether they are identical or not). Analysis of GCOD data that is presented on the website is performed in R by direct system calls.

## Utility and Discussion

**Figure 3:**
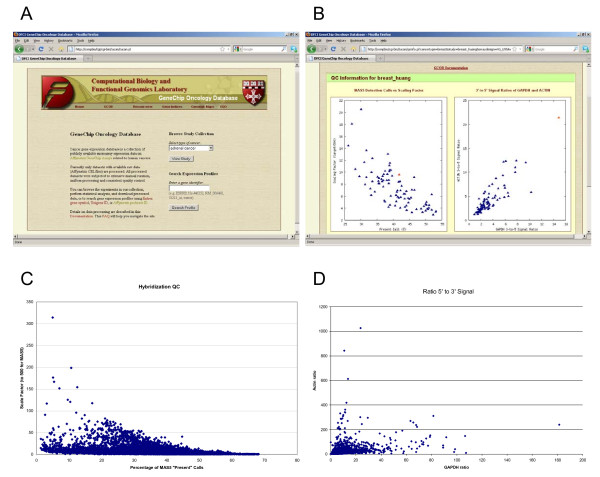
**GCOD Screenshots and QC Analysis**. A. The GCOD web site main page. B. A representative view of the QC information available on the GCOD site. C. Assessment of MAS5 Present/Absent call and scaling factor data. D. Display of the GAPDH and β-ACTIN 5' to 3' probe signal ratios.

Although the database provides overall organization of the information we have compiled, the most important aspect of GCOD is its presentation of those data to the end-users. We developed a series of web-based tools to allow access to the data based on use cases representing common questions users ask of expression data (Figure 3A). Study-centered views allow users to browse the individual studies, check the quality of hybridizations, download the processed data, and perform preliminary data analysis online. Gene-centered views allow users to query the expression profiles across multiple datasets. The integration of the TGI Resourcerer database [7] provides up-to-date annotation of the array probes and facilitates cross-comparison between the various Affymetrix array platforms.

### Study-centered views

The study-centered views allow users to browse the list of published studies and to search for datasets of interest. For each individual study the title of the publication, a summary of samples and experimental factors involved, and the total number of hybridizations on the specified array type are displayed. Listed next to each study name are three separate options: access QC information, compare experimental groups using a t-test, and download the dataset. The QC information (Figure 3B) includes two scatter plots showing the quality control information derived from the MAS 5.0 algorithm; one is the percentage of 'Present' calls vs. scaling factor (target = 500) and the other is the 3' to 5' signal ratios of the control transcripts GAPDH versus β-ACTIN [8]; both plots identify questionable hybridizations as outliers in the graphs.

To provide basic utilities for comparison between phenotypic groups defined by the curated sample annotation, we implemented Student's t-test with data-trimming filters, p-value thresholds for significance, and Bonferroni correction for multiple testing. Results are presented in a table that lists significant genes together with group means, standards deviations, differences between the means, degree of freedom and raw (and corrected) p-values. The results are sorted by p-value and each probeset name is linked to expression values for all the samples in our database. Users can browse the results and download them as a text file.

The download function offers users options to choose either MAS 5.0 or RMA normalized data, whether or not to exclude the data points from questionable hybridizations flagged by the QC filter, and whether the data should be trimmed by removal of data (rows) with less than the specified percentage of MAS 5.0 'Present' calls across samples (columns). Sample annotations classified by experimental factors will be listed on the header section of the downloaded data table with an option to arrange the columns by any user-chosen experimental factor. In addition, RMA normalized expression data, in the format of the BioConductor eSet data object, can be downloaded with the "R Data Download" link.

### Gene-centered views

**Figure 4:**
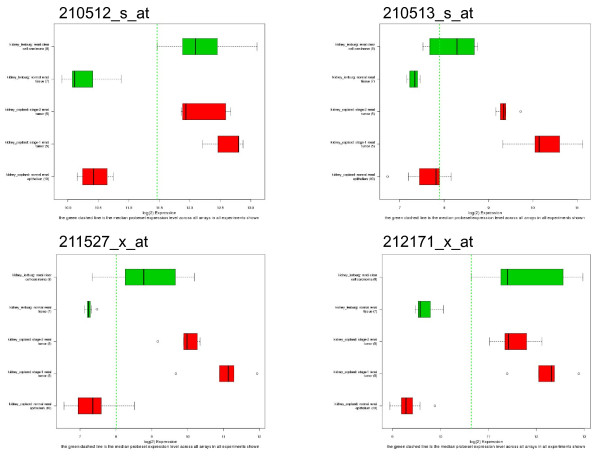
**Expression of VEGF in Kidney cancer**. Data from the expression as reported by probesets 210512_s_at (A), 210513_s_at (B), 211571_x_at (C), and 212171_x_at (D) in two studies of kidney cancer (9, 12). These reporters of VEGF gene expression show distinct gene expression differences in tumor vs. normal sample groups (top to bottom): clear cell carcinoma (Lenburg et al.), normal renal tissue (Lenburg et al.), stage 2 renal tumor (Copland, et al.), stage 1 renal tumor (Copland, et al.), normal renal epithelium (Copland, et al.).

**Figure 5:**
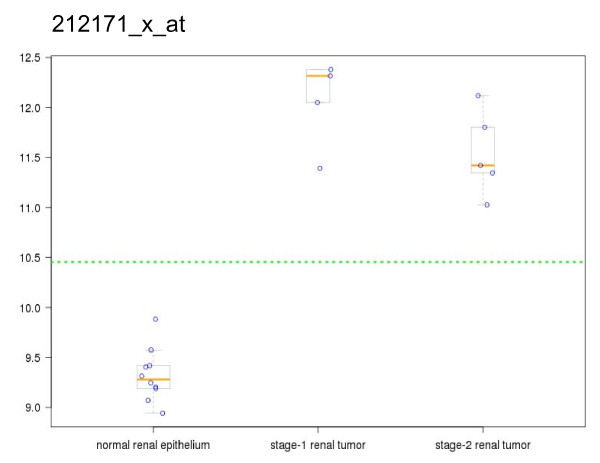
**Expression data from Probeset 212171_x_at**. RMA normalized and scaled signal data for probeset 212171_x_at in kidney tumor vs. normal tissue samples from Copland et al. (9). Box and whisker plots are overlaid with individual data points from the sample groups listed in the legend for Figure 4.

A common GCOD use case involves comparing the expression of a single gene across a large number of samples. Gene-centric searches allow users to query the database using any of the following gene-specific identifiers including gene symbol, a range of gene names and synonyms, GenBank accession number, UniGene id, RefSeq accession, Affymetrix probeset id, LocusLink identifier (equivalent to the Entrez Gene ID), or free text description. Lists of up to 300 identifiers may also be provided for batch searches (examples of entries for each identifier are shown in Table 2). These identifiers are automatically mapped to probeset ids, which are the primary identifiers used for expression measures. Graphical displays of expression values across all samples are presented as box plots which depict normalized expression results on a study-by-study basis (Figure 4). Box plots showing contrasting expression between the primary experimental subgroups (described in the corresponding published manuscript) can be generated as well (figure 5).

**Table 2:** Example Gene Identifier Entries for Gene-Centric Searches

Identifier	Example	List
Probeset	33218_at	33218_at, 1802_s_at, 216836_s_at, 210930_s_at
Genbank#	NM_006468	NM_006468NM_004452
Locus Link/Entrez ID	2103	2103, 2064
Gene Symbol	ESRRB	ESRRB ERBB2 HER-2
UniGene ID	Hs.446352	Hs.446352, ERBB2, HER2, 33218_at
Ref_seq ID	NM_006468	NM_006468 NM_004452
Free text	tumor	tumor, estrogen receptor

Single entries may be entered as shown below. Lists may be entered as comma, space, tab or newline separated identifiers. Any of the listed identifier types can be used in combinations in a list.

As an example, we examined VEGF expression across all of the studies represented in GCOD. Figure 4 shows the expression of all four probesets on the HG-U133A array that are annotated as VEGF probes. For most cancers, there is little difference in expression for VEGF; however the renal cancer studies clearly show differential expression for VEGF (Figure 4). One can also look at individual probesets. Figure 5 shows the individual mean normalized expression values of probeset 212717_x_at (VEGF) as box plots for each sample grouping from the Copland kidney cancer study [9] (Figure 5). The normalized data values used in generating the box plots can be downloaded in each case.

The expression of VEGF appears decreased compared to normal kidney samples in the Corbin, et al. [10] study of Wilms' tumor. We believe these observations to be completely accurate based on published experimental work that describes decreased VEGF expression in Wilms' tumor [11] using RT-PCR. Compared to clear-cell renal cell carcinoma (ccRCC) (Lenburg et al. [12]), which is a highly vascularized tumor [13], Wilms' tumor is an early childhood nephroblastoma that is non-invasive; elevated VEGF would be expected in ccRCC, but not necessarily so in Wilms' tumor. The published data appear to support our expression-based observations that VEGF has elevated expression in ccRCC renal cancer.

**Figure 6:**
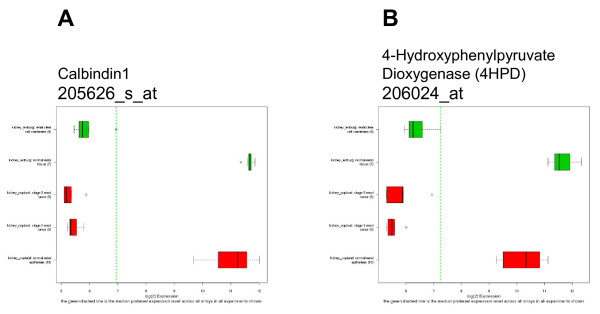
**Expression of CALB1 and 4-HPPD in Kidney cancer**. RMA normalized and scaled signal data for probesets 205626_s_at (CALB1) and 206024_at (4-HPPD) in two studies of kidney cancer (9, 12). The sample groups are listed in the legend for Figure 4.

In order to evaluate what other genes have altered expression in kidney cancer, we viewed the GCOD data 'by study' and selected one of the kidney cancer studies (Lenburg, et al. [12]). The top 20 entries from a t-test contrasting tumor versus normal samples (Table 3) from the Lenburg kidney cancer study shows several probesets other than VEGF that appear to have significant differences in their expression data. (The expression of VEGF is listed in these results, but is item 271 in the t-test result list.) We chose two for further examination. The expression of Calbindin1 (CALB1) and 4-Hydroxyphenylpyruvate dioxygenase (HPPD) are shown in Figure 6. Both genes are differentially repressed in these kidney cancer studies; their expression is opposite that of VEGF.

**Table 3:** Results from t-test contrasting normal versus tumor samples from the Lenburg renal cancer study

probeset	gene name	gene symbol	Adjusted p-value
205626_s_at	calbindin 1, 28kDa	CALB1	2.41E-08
216910_at			2.46E-08
206054_at	kininogen 1	KNG1	2.60E-08
219188_s_at	LRP16 protein	LRP16	2.64E-08
206024_at	4-hydroxyphenylpyruvate dioxygenase	HPD	6.79E-08
205243_at	solute carrier family 13 (sodium-dependent dicarboxylate transporter), member 3	SLC13A3	7.28E-08
221298_s_at	solute carrier family 22 (organic anion transporter), member 8	SLC22A8	9.68E-08
221605_s_at	pipecolic acid oxidase	PIPOX	9.72E-08
206716_at	uromodulin (uromucoid, Tamm-Horsfall glycoprotein)	UMOD	9.74E-08
205625_s_at	calbindin 1, 28kDa	CALB1	1.20E-07
204704_s_at			1.21E-07
209443_at	serine (or cysteine) proteinase inhibitor, clade A (alpha-1 antiproteinase, antitrypsin), member 5	SERPINA5	1.47E-07
206457_s_at	deiodinase, iodothyronine, type I	DIO1	1.61E-07
221590_s_at	aldehyde dehydrogenase 6 family, member A1	ALDH6A1	2.05E-07
206484_s_at	X-prolyl aminopeptidase (aminopeptidase P) 2, membrane-bound	XPNPEP2	2.19E-07
216092_s_at	solute carrier family 7 (cationic amino acid transporter, y+ system), member 8	SLC7A8	3.27E-07
202752_x_at	solute carrier family 7 (cationic amino acid transporter, y+ system), member 8	SLC7A8	3.75E-07
204254_s_at	vitamin D (1,25- dihydroxyvitamin D3) receptor	VDR	4.62E-07
205983_at	cyclin-dependent kinase (CDC2-like) 10	CDK10	5.10E-07
218844_at	hypothetical protein FLJ20920	FLJ20920	5.65E-07
[...]			
210512_s_at	vascular endothelial growth factor	VEGF	7.22E-03

The kidney_lenburg study was selected from GCOD. Then t-test was performed with the 2 sample groups; low-quality hybridizations and hybridizations with less than 5% Present calls were excluded from the t-test. Also, the Bonferonni correction for multiple testing was applied. Only the top 20 results are shown from a list of 308 results returned. Item 271 (VEGF) also is listed.

Calbindin-D_28k_, CALB1, is a vitamin D dependent, calcium binding protein that is expressed in several tissues including the kidney, pancreas and brain [14]. CALB1 acts to buffer calcium concentration in the blood and tissues, and may have regulatory properties similar to other calcium binding proteins, e.g. calmodulin and troponin-C. HPPD is part of the tyrosine catabolic pathway; it converts 4-hydroxyphenylpyruvate to homogentistate which is subsequently catabolyzed to acetoacetate and fumarate. HPPD is expressed in the liver and kidneys, as well as cerebral cortex, cerebellum and hippocampus. Mutations in HPPD result in type III tyrosinemia, a hereditary condition in which mild mental retardation and seizures occur due to the accumulation of tyrosine and phenylalanine in the blood. Oddly enough, inactivation of or deletion of HPPD alleviates the effects of type I tyrosinemia caused by deficiency of fumarylacetoacetase (the last enzyme in the tyrosine catabolism pathway), and an accumulation of fumarylacetoacetate, succinylacetoacetate and derivatives [15,16].

### Data Assessment

The current release of GCOD includes 125 studies consisting of a total of 13,591 hybridizations with data collected on 15 different Affymetrix GeneChip types as summarized in Table 4. The studies have an average of 4 experimental factors per study, and a modal value of 2 (±2.00, based on a Poisson distribution) and a maximum number of 30 experimental factors (lymphoma_hummel). Twenty-eight studies have only a single experimental factor. There are 198 different experimental factors assigned to the 125 studies in GCOD; the experimental factor "disease_state" is used most often, in 110 studies.

**Table 4:** Number of Arrays in GCOD Grouped by Array Platform

Array Type	Number of Arrays
HG-U133A	4815
HG-U133_Plus_2	2657
HG_U95Av2	2064
HG-U133B	1302
Hu6800	1034
HG_U95A	506
Hu35KsubA	257
HG_U95B	194
HG_U95C	193
U133AAofAv2	130
HG_U95D	128
HG_U95E	128
HG-U133A_2	105
HG-Focus	61
HC_G110	23

Total	13597

QC filters identify potentially poor hybridizations (Figures 3C and 3D) based on criteria that include: a) scaling factor values greater than 100, b) actin_ratio greater than 10 and gapdh_ratio greater than 10, and c) present call (detection) percentage less than 10. Hybridizations failing to meet these criteria are flagged for exclusion, but only excluded if the user selects the option to do so. Hybridizations failing to meet those criteria represent a) 0.124%, b) 3.70% and c) 1.94% of the data, respectively, with 5.63% of the hybridizations, in total, that fail to meet one or more of these criteria.

## Conclusion

The GCOD web site provides access to normalized and scaled gene expression data from analyses of a variety of cancer types. The site provides filtering based on QC analysis of the data, and the ability to do t-tests based on the experimental parameters for the individual studies in the database. The GCOD site also offers the option to download data for each study. In the near future we plan to augment the GCOD web site to include: a) additional data QC metrics, b) a cancer gene signatures search function, and c) a batch search function. Lastly, new data sets are added to GCOD as they become available.

## Availability

The data in GCOD is freely accessible at http://compbio.dfci.harvard.edu/gcod

**Supplementary information**: supplementary data are available at http://compbio.dfci.harvard.edu/gcod

## Authors' contributions

The GCOD web site was designed and initially implemented by FL based on discussions with JQ. FL downloaded, curated and processed many of the data sets that were initially loaded into the GCOD database. The databases were designed and built by JW, who wrote the ETL scripts currently in use to process data and load the databases. JW assisted with curation and processing of the most recently added data sets. The current web site implementation, all of the Perl/CGI scripts and all modifications to its design have been maintained by CA. DG acquired and curated about a third of the data sets, assisted with sample annotation validation of many of the data sets, and performed RMA processing for some data sets. JQ provided initial guidance and critical design decisions during the development of the GCOD web site and its use cases.   All authors read and approved the final manuscript.

## Supplementary Material

Additional file 1 **List of Data Sets Contained in the GCOD**. Characteristics of the data sets available in GCOD. The study name is a concatenation of the tumor type and the publication first author's name. Some studies have no available PubMed ID. Note: several studies include multiple ArrayDesign types and occupy more than one row in the table below Click here for file
