# Supplementary material for: GCOD - GeneChip Oncology Database
Source: BMC Bioinformatics. 2011 Feb 3;12:46. doi: 10.1186/1471-2105-12-46 (PMC3045303; doi:10.1186/1471-2105-12-46)
Supplement: Additional file 1 — List of Data Sets Contained in the GCOD. Characteristics of the data sets available in GCOD. The study name is a concatenation of the tumor type and the publication first author's name. Some studies have no available PubMed ID. Note: several studies include multiple ArrayDesign types and occupy more than one row in the table below [file 1471-2105-12-46-S1.DOC]

**Supplement Table 1: List of Data Sets Contained in the GCOD**

-------------------------------------------------------------------------------------------------------------------------------------

Characteristics of the data sets available in GCOD. The study name is a concatenation of the tumor type and the publication first author’s name. Some studies have no available PubMed ID. Note: several studies include multiple ArrayDesign types and occupy more than one row in the table below.

-------------------------------------------------------------------------------------------------------------------------------------

| Study Name | Tumor Type | PubMed ID | Hyb Count | | ArrayDesign | |
| --- | --- | --- | --- | --- | --- | --- |
|  |  |  |  |  | |  |
| adrenal_giordano | adrenal cancer | 12547710 | 19 |  | | HG_U95Av2 |
| adrenal_reynies | adrenal cancer |  | 92 |  | | HG-U133_Plus_2 |
| adrenal_west | adrenal cancer | 12547710 | 31 |  | | HG-U133A |
| bladder_dyrskjot | bladder cancer | 12469123 | 71 |  | | Hu6800 |
| bladder_dyrskjot1 | bladder cancer | 15173019 | 60 |  | | HG-U133A |
| bladder_stransky | bladder cancer | 17099711 | 103 |  | | HG_U95A |
| brain_dePreter | brain cancer | 16989664 | 24 |  | | HG-U133A |
| brain_dong | brain cancer | 16254489 | 18 |  | | HG-U133A |
| brain_fattet | brain cancer | 19197950 | 40 |  | | HG-U133_Plus_2 |
| brain_gutmann | brain cancer | 11929829 | 3 |  | | HG_U95A |
| brain_gutmann | brain cancer | 11929829 | 13 |  | | HG_U95Av2 |
| brain_khatua | brain cancer | 12702575 | 15 |  | | HG_U95Av2 |
| brain_kool | brain cancer | 18769486 | 62 |  | | HG-U133_Plus_2 |
| brain_macdonald | brain cancer | 11544480 | 23 |  | | HC_G110 |
| brain_macdonald2 | brain cancer | GSE3185 | 20 |  | | HG_U95Av2 |
| brain_margareto | brain cancer | 17873288 | 16 |  | | HG-U133A_2 |
| brain_mcardle | brain cancer | 15090470 | 24 |  | | HG-U133A |
| brain_murat | brain cancer | 18565887 | 84 |  | | HG-U133_Plus_2 |
| brain_nutt | brain cancer | 12670911 | 50 |  | | HG_U95Av2 |
| brain_phillips | brain cancer | 16530701 | 201 |  | | HG-U133A |
| brain_pomeroy | brain cancer | 11807556 | 98 |  | | Hu6800 |
| brain_rickman | brain cancer | 11559565 | 51 |  | | Hu6800 |
| brain_thompson | brain cancer | 16567768 | 46 |  | | HG-U133A |
| brain_turkheimer | brain cancer | 17140431 | 30 |  | | HG-U133_Plus_2 |
| brain_wang | brain cancer |  | 102 |  | | HG_U95A |
| breast_boersma | breast cancer |  | 95 |  | | HG-U133A |
| breast_chang | breast cancer | 12907009 | 24 |  | | HG_U95Av2 |
| breast_chen | breast cancer |  | 185 |  | | HG-U133_Plus_2 |
| breast_chin | breast cancer | 17157792 | 130 |  | | U133AAofAv2 |
| breast_farmer | breast cancer | 15897907 | 49 |  | | HG-U133A |
| breast_fournier | breast cancer | 16849555 | 12 |  | | HG-U133A |
| breast_huang | breast cancer | 12747878 | 89 |  | | HG_U95Av2 |
| breast_ivshina | breast cancer | 17079448 | 289 |  | | HG-U133A |
| breast_ivshina | breast cancer | 17079448 | 289 |  | | HG-U133B |
| breast_loi | breast cancer | 17401012 | 327 |  | | HG-U133A |
| breast_loi | breast cancer | 17401012 | 327 |  | | HG-U133B |
| breast_loi | breast cancer | 17401012 | 87 |  | | HG-U133_Plus_2 |
| breast_loi1 | breast cancer |  | 77 |  | | HG-U133_Plus_2 |
| breast_lu | breast cancer | 18297396 | 129 |  | | HG-U133_Plus_2 |
| breast_miller | breast cancer | 16141321 | 251 |  | | HG-U133A |
| breast_miller | breast cancer | 16141321 | 251 |  | | HG-U133B |
| breast_minn | breast cancer | 16049480 | 121 |  | | HG-U133A |
| breast_pawitan | breast cancer | 16280042 | 318 |  | | HG-U133A |
| breast_richardson | breast cancer | 16473279 | 47 |  | | HG-U133_Plus_2 |
| breast_schmidt | breast cancer |  | 200 |  | | HG-U133A |
| breast_seitz | breast cancer |  | 26 |  | | HG-U133A |
| breast_turashvili | breast cancer |  | 30 |  | | HG-U133_Plus_2 |
| breast_wang | breast cancer |  | 286 |  | | HG-U133A |
| breast_west | breast cancer | 11562467 | 49 |  | | Hu6800 |
| cervical_bachtiary | cervical cancer | 17020965 | 33 |  | | HG-U133_Plus_2 |
| cervical_scotto | cervical cancer |  | 66 |  | | HG-U133A |
| colon_ancona | colon cancer |  | 47 |  | | HG-U133A |
| colon_groene | colon cancer | 16721809 | 36 |  | | HG-U133_Plus_2 |
| colon_jorissen | colon cancer |  | 155 |  | | HG-U133_Plus_2 |
| colon_laiho | colon cancer | 16819509 | 37 |  | | HG-U133A |
| colon_staub | colon cancer |  | 62 |  | | HG-U133A |
| endometrium_wu | endometrium | 16355216 | 5 |  | | HG_U95A |
| endometrium_wu | endometrium | 16355216 | 13 |  | | HG_U95Av2 |
| esophagus_kimchi | esophagus cancer | 15833844 | 24 |  | | HG-U133A |
| germcell_korkola | germ cell cancer | 15870693 | 107 |  | | HG-U133A |
| germcell_korkola | germ cell cancer | 15870693 | 107 |  | | HG-U133B |
| headandneck_colella | head and neck cancer | E-MEXP-44 | 33 |  | | HG_U95Av2 |
| headandneck_colella | head and neck cancer | E-MEXP-44 | 35 |  | | Hu6800 |
| headandneck_cromer | head and neck cancer | | 18 |  | | HG_U95A |
| headandneck_cromer | head and neck cancer | | 20 |  | | HG_U95Av2 |
| headandneck_kuriakose | head and neck cancer | 15170515 | 44 |  | | HG_U95Av2 |
| headandneck_odonnell | head and neck cancer | 15558013 | 27 |  | | HG-U133A |
| headandneck_toruner | head and neck cancer | 15381369 | 20 |  | | HG-U133A |
| kidney_copland | kidney cancer | GSE6344 | 20 |  | | HG-U133A |
| kidney_copland | kidney cancer | GSE6344 | 20 |  | | HG-U133B |
| kidney_lenburg | renal cancer | 14641932 | 17 |  | | HG-U133A |
| kidney_lenburg | renal cancer | 14641932 | 17 |  | | HG-U133B |
| leukemia_armstrong | leukemia | 11731795 | 42 |  | | HG_U95A |
| leukemia_armstrong | leukemia | 11731795 | 30 |  | | HG_U95Av2 |
| leukemia_auer | leukemia |  | 48 |  | | HG-U133A |
| leukemia_cheok | leukemia | 12704389 | 10 |  | | HG_U95A |
| leukemia_cheok | leukemia | 12704389 | 110 |  | | HG_U95Av2 |
| leukemia_crossman | leukemia | 15820940 | 28 |  | | HG_U95Av2 |
| leukemia_ferrando | leukemia | 12086890 | 39 |  | | Hu6800 |
| leukemia_haslinger | leukemia | 15459216 | 28 |  | | HG_U95A |
| leukemia_haslinger | leukemia | 15459216 | 83 |  | | HG_U95Av2 |
| leukemia_holleman | leukemia | 15295046 | 173 |  | | HG-U133A |
| leukemia_lughart | leukemia | 15837626 | 129 |  | | HG-U133A |
| leukemia_mcelwaine | leukemia | 15180862 | 20 |  | | HG-U133A |
| leukemia_neumann | leukemia | 15618956 | 14 |  | | HG-Focus |
| leukemia_ross1 | leukemia | 12730115 | 132 |  | | HG-U133A |
| leukemia_ross1 | leukemia | 12730115 | 132 |  | | HG-U133B |
| leukemia_ross2 | leukemia | 15226186 | 155 |  | | HG-U133A |
| leukemia_soulier | leukemia | 15774621 | 104 |  | | HG-U133A |
| leukemia_stegmaier | leukemia | 14770183 | 87 |  | | HG-U133A |
| leukemia_stegmaier | leukemia | 14770183 | 30 |  | | Hu6800 |
| leukemia_teuffel | leukemia | 15257931 | 31 |  | | HG-U133A |
| leukemia_yeoh | leukemia | 12086872 | 335 |  | | HG_U95Av2 |
| lung_beer | lung cancer | 12118244 | 96 |  | | Hu6800 |
| lung_bhattacharjee | lung cancer | 11707567 | 254 |  | | HG_U95Av2 |
| lung_chao | lung cancer |  | 4 |  | | HG-U133_Plus_2 |
| lung_ding | lung cancer |  | 75 |  | | HG-U133_Plus_2 |
| lung_gordon | lung cancer | 12208747 | 33 |  | | HG_U95Av2 |
| lung_lu | lung cancer | 17194181 | 18 |  | | HG-U133A |
| lung_lu | lung cancer | 17194181 | 18 |  | | HG-U133B |
| lung_lu | lung cancer | 17194181 | 36 |  | | HG_U95Av2 |
| lung_rohr | lung cancer | GSE6044 | 47 |  | | HG-Focus |
| lung_yap | lung cancer | 15653641 | 58 |  | | HG-U133A |
| lymphoma_hummel | lymphoma | 16760442 | 221 |  | | HG-U133A |
| lymphoma_piccaluga | lymphoma | 17304354 | 60 |  | | HG-U133_Plus_2 |
| lymphoma_shipp | lymphoma | 11786909 | 77 |  | | Hu6800 |
| multicancer_ramaswamy | multicancer | 11742071 | 257 |  | | Hu35KsubA |
| multicancer_ramaswamy | multicancer | 11742071 | 254 |  | | Hu6800 |
| multiplemyeloma_agnelli | multiple myeloma | 17367409 | 102 |  | | HG-U133A |
| multiplemyeloma_agnelli1 | multiple myeloma | 17229636 | 90 |  | | HG-U133A |
| nci60_hgu133 | nci60 | 17339364 | 59 |  | | HG-U133A |
| nci60_hgu133 | nci60 | 17339364 | 59 |  | | HG-U133B |
| nci60_hgu95 | nci60 | 17339364 | 60 |  | | HG_U95A |
| nci60_hgu95 | nci60 | 17339364 | 60 |  | | HG_U95B |
| nci60_hgu95 | nci60 | 17339364 | 60 |  | | HG_U95C |
| nci60_hgu95 | nci60 | 17339364 | 60 |  | | HG_U95D |
| nci60_hgu95 | nci60 | 17339364 | 60 |  | | HG_U95E |
| nci60_hu6800 | nci60 | 11553813 | 60 |  | | Hu6800 |
| normal_ge | normal tissue | 15950434 | 36 |  | | HG-U133A |
| normal_su | normal tissue | 11904358 | 84 |  | | HG_U95A |
| ovary_hendrix | ovarian cancer | 16452189 | 103 |  | | HG-U133A |
| ovary_jochumsen | ovarian cancer | 17367315 | 27 |  | | HG-U133_Plus_2 |
| ovary_lancaster | ovarian cancer | 14706684 | 34 |  | | Hu6800 |
| ovary_marquez | ovarian cancer |  | 68 |  | | HG_U95Av2 |
| ovary_marquez | ovarian cancer |  | 68 |  | | HG_U95B |
| ovary_marquez | ovarian cancer |  | 68 |  | | HG_U95C |
| ovary_marquez | ovarian cancer |  | 68 |  | | HG_U95D |
| ovary_marquez | ovarian cancer |  | 68 |  | | HG_U95E |
| ovary_schwartz | ovarian cancer | 12183431 | 113 |  | | Hu6800 |
| ovary_tone | ovarian cancer |  | 37 |  | | HG-U133_Plus_2 |
| ovary_tothill | ovarian cancer |  | 285 |  | | HG-U133_Plus_2 |
| pancreas_grutzmann | pancreas cancer | 15548371 | 26 |  | | HG-U133A |
| pancreas_grutzmann | pancreas cancer | 15548371 | 25 |  | | HG-U133B |
| pancreas_logston | pancreas cancer | 12750293 | 27 |  | | Hu6800 |
| prostate_best | prostate cancer | 16203770 | 20 |  | | HG-U133A |
| prostate_chandran | prostate cancer | 17430594 | 65 |  | | HG_U95Av2 |
| prostate_chandran | prostate cancer | 17430594 | 66 |  | | HG_U95B |
| prostate_chandran | prostate cancer | 17430594 | 65 |  | | HG_U95C |
| prostate_latulippe | prostate cancer | 12154061 | 35 |  | | HG_U95A |
| prostate_liu | prostate cancer |  | 57 |  | | HG-U133A |
| prostate_liu | prostate cancer |  | 57 |  | | HG-U133B |
| prostate_singh | prostate cancer | 12086878 | 102 |  | | HG_U95Av2 |
| prostate_traka | prostate cancer | 18596959 | 81 |  | | HG-U133_Plus_2 |
| prostate_tsavachidou | prostate cancer | 19244175 | 85 |  | | HG-U133A |
| prostate_varambally | prostate cancer | 16286247 | 19 |  | | HG-U133_Plus_2 |
| prostate_wallace | prostate cancer | 18245496 | 89 |  | | HG-U133A_2 |
| prostate_wang | prostate cancer | 17001317 | 148 |  | | HG-U133A |
| prostate_xia | prostate cancer |  | 88 |  | | HG_U95Av2 |
| prostate_yu | prostate cancer | 15254046 | 492 |  | | HG_U95Av2 |
| sarcoma_schafer | sarcoma | 15313887 | 30 |  | | HG-U133A |
| sarcoma_wachtel | sarcoma | 15313887 | 30 |  | | HG-U133A |
| Thyroid_huang | thyroid cancer | 11752453 | 16 |  | | HG_U95A |
| Thyroid_reyes | thyroid cancer | GSE3678 | 14 |  | | HG-U133_Plus_2 |
| Thyroid_sarquis | thyroid cancer | 16249278 | 24 |  | | HG-U133A |
| Thyroid_vasko | thyroid cancer | 17296934 | 18 |  | | HG-U133_Plus_2 |
| Thyroid_weber | thyroid cancer | 15713710 | 24 |  | | HG-U133A |
